# Heart Failure and Wide QRS: Clinical and Pharmacological Perspectives

**DOI:** 10.3390/biomedicines13061462

**Published:** 2025-06-13

**Authors:** Alfredo Mauriello, Adriana Correra, Gerardo Elia Del Vecchio, Martina Grieco, Arianna Amata, Pierpaolo Di Micco, Egidio Imbalzano, Mariano Paternoster, Antonia Ascrizzi, Vincenzo Quagliariello, Nicola Maurea, Francesco Giallauria, Antonello D’Andrea, Vincenzo Russo

**Affiliations:** 1 S.C. Cardiologia, Istituto Nazionale Tumori, IRCCS, Fondazione “G. Pascale”, 80131 Naples, Italy; v.quagliariello@istitutotumori.na.it (V.Q.); n.maurea@istitutotumori.na.it (N.M.); 2 Intensive Cardiac Care Unit, San Giuseppe Moscati Hospital, ASL Caserta, 81031 Aversa, Italy; adrianacorrera@gmail.com; 3 Cardiology Unit, “A. Guerriero” Hospital, ASL Caserta, 81025 Marcianise, Italy; delvecchiogerardo@gmail.com; 4 Cardiology Unit, S. Giovanni Bosco Hospital, ASL Napoli 1, 80100 Naples, Italy; m.grieco93@gmail.com; 5 Department of Cardiovascular Medicine, Fondazione Policlinico Universitario A. Gemelli, IRCCS, 00168 Rome, Italy; arianna.amata2@gmail.com; 6 Department of Medicine, Buon Consiglio Fatebenefratelli Hospital of Naples, 80122 Naples, Italy; pdimicco@libero.it; 7 Dipartimento Di Clinica Medica E Farmacologia, University of Messina, 98100 Messina, Italy; egidio.imbalzano@unime.it; 8 Department of Advanced Biomedical Sciences, School of Medicine, University of Naples Federico II, 80131 Naples, Italy; marianopaternoster@unina.it; 9 Cardiology Unit, Department of Medical and Translational Sciences, University of Campania “Luigi Vanvitelli”, Monaldi Hospital, 80131 Naples, Italy; antonia.ascrizzi@gmail.com (A.A.); vincenzo.russo@unicampania.it (V.R.); 10 Department of Translational Medical Sciences, “Federico II” University of Naples, Via S. Pansini 5, 80131 Naples, Italy; francesco.giallauria@unina.it; 11 Cardiology and Intensive Care Unit, Department of Cardiology, Umberto I Hospital, 84014 Nocera Inferiore, Italy

**Keywords:** heart failure, sacubitril/valsartan, dapagliflozin, empagliflozin, vericiguat, ventricular dyssynchrony, QRS widening, cardiac remodeling

## Abstract

Heart failure (HF) is a highly prevalent cardiovascular clinical syndrome. Health care spending on HF treatment is high. Therefore, its treatment has generated a great deal of interest in pharmacological research in recent years. Recent guidelines have introduced several molecules for the treatment of HF that have demonstrated safety, and above all, efficacy. One of the worst aspects of HF is ventricular dyssynchrony (VD) with a wide QRS interval. Currently, the cornerstone of VD therapy is cardiac resynchronization therapy (CRT). Our comprehensive review aims to analyze the effects of new molecules on QRS width and understand whether these molecules can provide benefits.

## 1. Introduction

Heart failure (HF) is a clinical syndrome characterized by typical symptoms (e.g., breathlessness, ankle swelling, and fatigue) that may be associated with signs (e.g., elevated jugular venous pressure, pulmonary crackles, and peripheral oedema) caused by a structural and/or functional cardiac abnormality and resulting in reduced cardiac output (CO) and/or elevated intracardiac pressures at rest or during stress [1,2]. In Europe, the HF incidence is approximately 3–5 per 1000 person-years. The HF prevalence is approximately 1–2% in adults, and it increases with age, accounting for up to 10% in patients aged 70 years or over [1]. The new European Society of Cardiology (ESC) classification, which includes HF with preserved (HFpEF) and mid-range (HFmrEF) ejection fractions, is expected to result in an increased diagnostic prevalence of approximately 50% worldwide [3].

**Figure 1:**
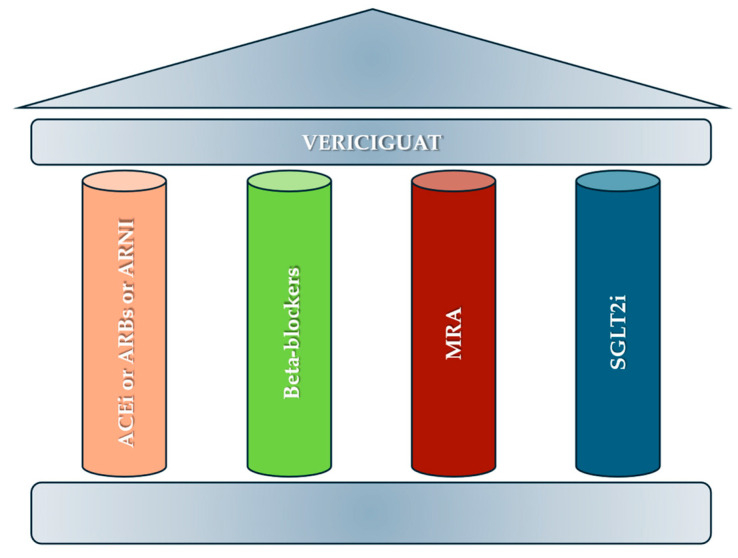
The five pillars of therapy for the pharmacological treatment of chronic heart failure. ACEi: angiotensin-converting enzyme inhibitors; ARBs: angiotensin-receptor blockers; ARNI: angiotensin receptor neprilysin inhibitor; MRA: mineralocorticoid receptor antagonist; SGLT2i: sodium glucose co-transporter 2 inhibitors.

Ventricular dyssynchrony (VD) is the dyssynchronous contraction and relaxation of the left and right ventricles [4]. VD can result from various disturbances in the electrical conduction of the heart, such as left bundle branch block (LBBB) or right bundle branch block (RBBB), and can lead to a reduction in the ejection fraction of the left ventricle (LV), contributing to the development of HF. Initially, electrocardiographic parameters, particularly the duration of the QRS complex (>120 milliseconds), were used to study VD. In recent years, however, it has emerged that QRS duration is not a reliable indicator of VD [5]. To overcome the limitations of the ECG, mechanical parameters of dyssynchrony have been proposed, usually assessed by echocardiographic techniques [5,6]. The results published so far indicate that mechanical dyssynchrony is a better predictor of outcome than QRS duration, although the optimal echocardiographic parameter for assessing dyssynchrony has not yet been determined [6]. VD is a frequently observed feature in patients with HF and is recognized as an essential predictor of worse outcomes when untreated [7]. The prevalence of VD in patients with HF varies according to the diagnostic criteria used. Using electrocardiographic criteria, approximately 30–50% of patients with HF have VD, identified by a QRS ≥ 120 ms on the ECG. Using advanced echocardiographic criteria, such as tissue Doppler imaging or speckle tracking, the prevalence may be higher, even up to 50–60% [8]. The presence of VD leads to inefficient LV contraction with a decreased CO [9]. Moreover, patients with VD are at an increased risk of adverse cardiac events [10]. The optimal treatment of HFrEF and HFmrEF encompasses the five pillars of pharmacological therapies, including angiotensin-converting enzyme inhibitors (ACEi) or angiotensin receptor blockers (ARBs), beta-blockers, and a mineralocorticoid receptor antagonist (MRA). Recently, novel drugs have been introduced, such as sodium–glucose cotransporter 2 inhibitors (SGLT2i), the angiotensin receptor neprilysin inhibitor (ARNI), and vericiguat (Figure 1) [2]. We have conducted a comprehensive review that aims to evaluate the hemodynamic impact of VD and the effects of recent HF drugs on VD.

## 2. Intraventricular Conduction Delay in Heart Failure: Pathophysiological Mechanisms

HF is a clinical syndrome defined by the heart’s inability to pump sufficient blood to meet the body’s metabolic demands, or by doing so only at the expense of elevated filling pressures [1,11]. HF triggers a cascade of compensatory mechanisms that, while initially beneficial, ultimately become maladaptive. A central feature of this process is neurohormonal activation, in which peripheral hypoperfusion stimulates both the sympathetic nervous system (SNS) and the renin–angiotensin–aldosterone system (RAAS). Although these responses help sustain perfusion in the short term, chronic activation leads to vasoconstriction, fluid retention, and increased afterload, further exacerbating cardiac stress. Over time, sustained neurohormonal stimulation promotes myocardial remodeling, including fibrosis and structural changes that stiffen the ventricular wall, impair both relaxation and contraction, and drive the progressive functional decline that is characteristic of chronic HF [12].

Cardiac remodeling refers to the structural, functional, cellular, and molecular changes that occur in the heart [13]. This could be caused by direct myocardial injury, such as in myocardial infarction, myocarditis, or other cardiomyopathies, or it may develop in response to hemodynamic stress, such as in cases of pressure or volume overload [14]. Remodeling can be adaptive (as in the athlete’s heart or pregnancy) or maladaptive, contributing to HF progression. Maladaptive remodeling involves myocyte hypertrophy, fibroblast proliferation, myocyte loss through apoptosis, and extracellular matrix changes, particularly increased collagen deposition, leading to fibrosis and impaired cardiac mechanics [15,16].

These changes, and particularly the development of myocardial fibrosis, affect not only systolic and diastolic function but also the genesis and propagation of electrical impulses, leading to conduction abnormalities and arrhythmias. Even if the exact mechanism remains unclear, myocardial fiber disarray and fibrosis of the conduction system are considered the main mechanisms that lead to conduction delay [17]. Additionally, ion channel dysfunction, altered calcium handling, rarefaction of gap junction proteins—specifically, connexins 40, 43, and 45—and autonomic nervous system imbalances further contribute to conduction abnormalities [18,19].

These alterations cause several conduction abnormalities in about one-third of HF patients [20,21]. Among them, LBBB and other non-specific diffuse intraventricular conduction delays were described in approximately 25% and 15% of patients with HF and HF with reduced ejection fraction (HFrEF), respectively [22,23].

Conduction delay and the subsequent abnormal mechanical activation and contraction lead to impaired LV systolic and diastolic function, abnormalities in myocardial metabolism and perfusion, exacerbation of mitral regurgitation, decline in RV systolic function, and increased arrhythmic risk [24,25].

**Figure 2:**
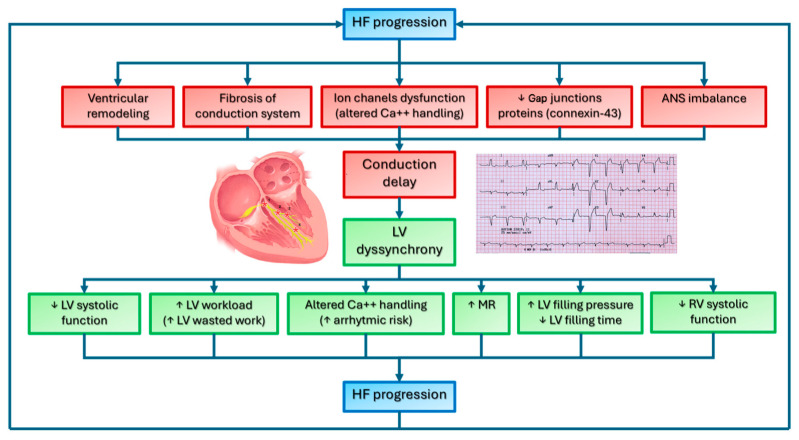
Pathophysiology of intraventricular conduction delay in heart failure. ANS: autonomic nervous system; HF: heart failure; LV: left ventricle; MR: mitral regurgitation; RV: right ventricle; ↑: increase; ↓: decrease.

This creates a vicious cycle in which progressive LV failure is often linked to worsening conduction abnormalities (Figure 2) [26].

### 2.1. Left Bundle Branch Block and Left Ventricle Systolic Function

In LBBB, LV activation usually occurs via the right bundle branch, causing right-to-left septal activation, which is opposite to the normal pattern. The LV lateral free wall is then activated through myocardial tissue, which conducts more slowly than the specialized Purkinje fibers [27]. The resulting abnormal regional motion impairs LV efficiency [28]. Indeed, early septal activation occurs when the LV lateral wall is relaxed, causing septal contraction to displace blood toward the stretched lateral wall, increasing its preload. When the lateral wall contracts, it displaces blood back to the septum, stretching and shifting toward the right ventricle. The septum absorbs energy from the lateral wall’s work, resulting in wasted energy in the septum [29]. As the lateral wall takes on a larger workload, hypertrophy often occurs while the septum thins. This imbalance contributes to adverse remodeling in HF patients with LBBB [24]. In a few patients, the last area to be activated may be the anterior or septal region [9]. As a result, regional wall contractions do not efficiently contribute to pressure buildup in the left ventricle but instead cause significant blood volume shifts within the LV cavity. This ultimately leads to reduced LV pumping efficiency, as the LV ejection fraction (EF) decreases despite maintained or even increased energy demand [9].

In a study simulating LBBB with sequential atrial and RV pacing, stroke volume (SV) decreased by 10%, while LV end-systolic volume increased by 15% [30]. VD can lead to a relative reduction in LV EF of approximately 20%. [20] Systolic dysfunction caused by conduction delay has also been demonstrated in patients with preserved LV EF. Indeed, global longitudinal strain (GLS), a more sensitive measure of systolic function than LV EF, was subnormal in a small study involving 11 asymptomatic patients with LBBB and normal LV EF. Additionally, when these patients were subjected to acute blood pressure elevation, they experienced a more significant reduction in LV EF compared to completely healthy individuals, suggesting impaired afterload tolerance [31].

### 2.2. Left Bundle Branch Block and Myocardial Metabolism

Such uncoordinated contraction raises myocardial energy demands and workload, as demonstrated with non-invasive myocardial work evaluation [32,33].

The septal segments contract early at low intraventricular pressure, generating minimal positive work, while the lateral wall contracts late at peak pressure, producing significantly more positive work. As the lateral wall shortens, the septal segments elongate, absorbing lateral wall-generated positive work, resulting in a characteristic figure-eight pressure–strain loop pattern [29,33,34,35]. Globally, septal segments present a total negative work in systole. Thus, septal contraction does not contribute to ventricular ejection, while the lateral wall compensates with extra work to maintain cardiac function. Interestingly, this workload disparity correlates with differences in metabolic activity, with early activated septal segments showing lower glucose demand and reduced myocardial blood flow and oxygen uptake [24,33,35,36].

When examined using nuclear imaging techniques, many patients with LBBB exhibit reduced septal perfusion despite the absence of obstructive coronary artery disease [37]. The exact mechanism of this finding is not entirely understood. However, two hypotheses exist. One proposed reason is that prolonged septal systole during exercise-induced tachycardia may contribute to this phenomenon, as myocardial perfusion occurs primarily during diastole [38]. However, the most likely explanation is the normal autoregulation of myocardial microcirculation, where perfusion decreases due to lower metabolic demand from reduced septal workload [25,33]. Septal hypoperfusion is thought to be the cause of the so-called “painful LBBB syndrome”, characterized by chest pain during intermittent LBBB, although the hypothesis is not confirmed [38]. Over time, prolonged abnormal activation triggers molecular and cellular remodeling, affecting protein expression and cardiomyocyte calcium handling [39,40]. Studies in animal models of dyssynchronous HF have shown alterations in Ca^2+^ dynamics, including changes in SERCA and PLB, and gap junction remodeling, particularly in the late-activated, high-stress left-ventricular free wall [41,42]. Dysfunctional calcium handling further promotes a pro-arrhythmic state [26,43].

### 2.3. Left Bundle Branch Block and Mitral Regurgitation

Conduction delay contributes to mitral regurgitation (MR) through several mechanisms that disrupt normal valve function [24]. In LBBB, the coordinated activation of papillary muscles and LV walls is disrupted, leading to a reduction in closing forces of the mitral valve (MV) apparatus, causing MR [9,44]. The impaired LV pressure rise in LBBB diminishes the force required for proper MV closure, thereby worsening MR. Additionally, LBBB exacerbates systolic dysfunction, leading to LV remodeling and lateral displacement of the papillary muscles [23]. When displaced laterally due to LV dilation, the papillary muscles exert lateral instead of vertical forces on the chordae, further impairing valve closure. This alters valve function, leading to “tenting”, where mitral leaflet tips move distally during systole, reducing coaptation and causing regurgitation [24,45].

### 2.4. Left Bundle Branch Block and Diastolic Function

Dyssynchrony in conduction delay also affects diastolic function [46,47,48,49]. In patients with LBBB, diastolic dysfunction appears to result from mechanical dyssynchrony that disrupts regular LV relaxation and filling. Studies have shown that LBBB is associated with a prolonged isovolumetric relaxation time, slower LV pressure decay, and a reduced rate of pressure fall, which collectively delay and shorten the diastolic filling phase [49,50,51]. These alterations reduce the overall left-ventricular (LV) filling time, potentially compromising stroke volume (SV), and require higher left-atrial pressures to maintain adequate preload [50]. Although these diastolic abnormalities are well documented, their direct contribution to HF symptoms in patients with isolated LBBB remains unclear and requires further investigation [24].

### 2.5. Effects of Left Bundle Branch Block on Right Ventricle Function

LBBB also seems to impair RV systolic function. This is mainly an indirect effect of LV dysfunction, worsened by VD, which leads to RV dysfunction through increased left-sided filling pressure [24,50]. This causes pulmonary congestion and pulmonary hypertension, which in turn raises RV afterload. However, abnormal septal motion during LBBB directly affects RV systolic function [52].

### 2.6. Right Bundle Branch Block in Heart Failure

Controversy exists regarding the impact of RBBB on ventricular function and metabolism, as well as regarding the benefits of CRT in these patients [53,54,55]. However, the presence of RBBB is linked to worse prognoses, particularly in the presence of underlying heart disease or associated conduction abnormalities [56,57]. Several studies have demonstrated the significant impact of RBBB on right and left ventricular function and metabolism. Indeed, RBBB causes delayed activation in the anterior and lateral walls of the RV, mirroring the pattern seen in the LV of LBBB patients [58,59,60]. Patients with RBBB exhibit larger RV dimensions and show RV mechanical dysfunction due to delayed free RV wall contraction, systolic dyssynchrony, and uncoordinated motion between the left and right ventricles, as demonstrated in studies using tissue Doppler imaging [61]. Additionally, RBBB seems to cause a prolonged pre-ejection period and isovolumic relaxation time, indicating that RBBB impairs not only RV systolic but also diastolic function through altered myocardial contractility and local remodeling, even if LV function remains substantially unaffected [61]. However, beyond the effects on RV, some studies have demonstrated that the presence of RBBB could also affect LV function. Indeed, RBBB could determine LV mechanical dyssynchrony, affecting the EF in a similar manner to patients with LBBB [62]. Moreover, RBBB increases LV mechanical dispersion, leading to impaired LV deformation and efficiency, as recently demonstrated using echocardiographic deformation parameters [63].

## 3. Sacubitril/Valsartan

Sacubitril/valsartan, a complex containing the neprilysin inhibitor sacubitril and the ARB valsartan, augments endogenous compensatory vasoactive peptides by inhibiting their breakdown, and, in addition, blocks the renin–angiotensin system [64].

The Prospective Comparison of Angiotensin Receptor Neprilysin Inhibitors (ARNIs) With Angiotensin Converting Enzyme Inhibitors (ACEI) to Determine Impact on Global Mortality and Morbidity in Heart Failure (PARADIGM-HF) trial enrolled 8442 HFrEF patients with a mean age of 63.8 ± 11.5 years [64].

In this double-blind trial, 8442 patients with New York Heart Association (NYHA) class II, III, or IV HF and an ejection fraction of 40% or less were randomly assigned to receive either sacubitril/valsartan (at a dose of 200 mg twice daily) or enalapril (at a dose of 10 mg twice daily), in addition to recommended therapy.

Compared with enalapril, sacubitril/valsartan reduced the risk of hospitalization for HF by 21% (*p* < 0.001) and decreased the symptoms and physical limitations of HF by 36% (*p* = 0.001).

No RCT sub-analyses explored the effects of sacubitril/valsartan among patients with HF and wide QRS; however, several observational studies have evaluated this correlation [65,66,67].

A study cohort was selected from the Treatment with Angiotensin Receptor Neprilysin Inhibitor for Taiwan Heart Failure (TAROT-HF) patient study, which is a principal investigator-initiated, multicenter, observational study of patients with HFrEF in Taiwan [67]. The TAROT-HF study includes clinical data, baseline electrocardiograms (ECGs), and baseline and serial follow-up echocardiograms from more than 1700 patients with HFrEF who received sacubitril/valsartan treatment at 10 hospitals between March 2017 and March 2021. Patients were classified as Group 1: ‘CRT eligible group’ if they presented LVEF ≤ 35% plus LBBB QRS morphology and QRS duration ≥ 130 ms or non-LBBB QRS morphology and QRS duration ≥ 150 ms at baseline ECG. Patients with LVEF ≤ 35% but who did not meet the ECG indications for CRT were classified as Group 2: ‘CRT ineligible group’. Those patients with baseline LVEF 35–40% and those who had already received CRT implantation before sacubitril/valsartan treatment were excluded from analysis. A total of 1524 patients, aged 65.8 ± 13.6 years, with a baseline LVEF ≤ 35% who initiated sacubitril/valsartan treatment, were enrolled. Propensity score matching was performed to adjust for confounders, and 1168 patients were analyzed. Baseline characteristics were comparable between the two groups (Groups 1 and 2). The improvements in LVEF and left-ventricular end-systolic volume index (LVESVi) were more significant in Group 2 than in Group 1 after 1 year of sacubitril/valsartan treatment (*p* < 0.001); LVEF improved to ≥50% in Groups 1 and 2, constituting 5.2% and 20.2% after 1 year of sacubitril/valsartan treatment (*p* < 0.001). Multivariate analyses showed that wide QRS durations were negatively associated with reverse cardiac remodeling in those patients with HFrEF receiving sacubitril/valsartan treatment.

A prospective study enrolled 100 patients (mean age 56.1, 8.2, 78% male) with non-ischemic dilated cardiomyopathy [66]. Before starting sacubitril/valsartan therapy, an ECG and echocardiogram were performed. Six months following the optimal dose, if LVEF improved by more than 5%, the patients were considered notable sacubitril/valsartan treatment responders. During follow-up, the QRS width was reduced from 123.7  ±  20.3 to 117.1  ±  18.8 ms (*p <* 0.001).

In addition, a study evaluated the effects of sacubitril/valsartan on the CRT non-responder population [68]. A prospective observational study included 190 CRT-D non-responder patients with symptomatic HFrEF despite receiving the optimal medical therapy for at least 1 year [68]. Sacubitril/valsartan add-on therapy in CRT-D non-responder patients is associated with 19.5% of additional responders, and a reduction in HF symptoms (*p* < 0.001) and rehospitalizations by 16% (*p* < 0.001), AF burden by 6% (*p* < 0.001), and ventricular arrhythmias by 15.3% (*p* < 0.001).

A retrospective study of 368 HFrEF patients treated with sacubitril/valsartan [65] showed that 56 (15%) had baseline LBBB. The mean age was 64 (±13.5 years), 53.6% were male, and 78.6% had non-ischemic cardiomyopathy. During a median (IQR) follow-up of 9.1 months (5–18 months), five (9%) resolved LBBB to normal conduction (*p* = 0.11), and two (4%) improved to non-specific intraventricular conduction delay (*p* = 0.08).

The beneficial effects of sacubitril/valsartan on VD could be explained by the potential role of sacubitril/valsartan in cardiac reverse remodeling, which may improve VD [69].

ARBs and ACEIs were not considered because the review focuses solely on new drugs used in HF.

## 4. SGLT2i

SGLT2i are a class of drugs that have as their main effect the blocking of the reabsorption of glucose in the early proximal tubule of the nephron, leading to increased glycosuria and a reduction in glycemia [2].

### 4.1. Role of Dapagliflozin in Wide QRS

The Dapagliflozin And Prevention of Adverse Outcomes in Heart Failure (DAPA-HF) trial enrolled 4744 patients with a mean age of 66 ± 11 years, NYHA class II, III, or IV HF, and an ejection fraction of 40% or less to receive either dapagliflozin (at a dose of 10 mg once daily) or placebo, in addition to recommended therapy.

A post hoc analysis [70] of the DAPA-HF trial [71] and Dapagliflozin Evaluation to Improve the Lives of Patients With Preserved Ejection Fraction Heart Failure (DELIVER) trial [72] has examined the relationship between dapagliflozin and QRS duration in HF. A total of 9824 patients with HF in the DAPA-HF and DELIVER trials were considered. Patients with longer QRS duration had lower LVEF, were more likely to be male, had a more extended history of HF, had higher N-terminal pro-b-type natriuretic peptide (NT-pro-BNP) levels, and had less atrial fibrillation (AF) [73,74]. Patients were divided into those with HFrEF and those with HFmrEF/HFpEF.

While a longer QRS interval was also associated with a higher risk of worsening HF in both HF phenotypes, QRS duration was only related to the risk of cardiovascular death (and all-cause death) in patients with HFrEF (QRS duration 120–149 ms—*p* = 0.001; QRS duration ≥ 150 ms—*p* = 0.009) and not in those with HFmrEF/HFpEF (QRS duration 120–149 ms—*p* = 0.98; QRS duration ≥ 150 ms—*p* = 0.13). The rates of the primary composite outcome were 9.2 (95% CI 8.7–9.7), 14.3 (13.0–15.7), and 15.9 (14.1–17.9) per 100 patient-years in the <120, 120–149, and ≥150 ms groups, respectively. These event rates were observed both in HFrEF and HFmrEF/HFpEF. In this post hoc analysis, dapagliflozin, compared with placebo, reduced the risk of the primary outcome consistently across the QRS duration subgroups (HR [95% CI] 0.75 [0.67–0.85], 0.79 [0.65–0.96], and 0.89 [0.70–1.13] in the <120, 120–149, and ≥150 ms groups, respectively; *p* for interaction = 0.28). The effect of dapagliflozin on the primary outcome was consistent across the QRS duration, regardless of HF phenotype, that is, HFrEF or HFmrEF/HFpEF. In conclusion, dapagliflozin reduced the QRS duration, but its ability to reduce the risk of the primary outcome was independent of QRS duration in DAPA-HF and DELIVER [70].

### 4.2. Role of Empagliflozin in Wide QRS

The Empagliflozin Outcome Trial in Patients With Chronic Heart Failure and a Reduced Ejection Fraction (EMPEROR-Reduced) enrolled 3730 patients with a mean age of 67 ± 11 years, with NYHA class II, III, or IV HF and an ejection fraction of 40% or less to receive empagliflozin (10 mg once daily) or placebo in addition to recommended therapy.

Regarding empagliflozin, there is only one retrospective study in the literature [75]. Of the 361 patients who received at least one prescription for empagliflozin during the study period, 101 had at least one ECG after initiation of empagliflozin. It was included in the study, which consisted of 50.5% female participants. Most patients (61.4%) were on the 10 mg daily dose; the remaining were on the 25 mg dose. An increase in several ECG metrics was observed after the initiation of empagliflozin, none of which were statistically significant: the PR interval increased by 4 ms above baseline (170 ± 22 versus 174 ± 32 ms, *p* = 0.06), the QRS duration increased by an average of 6 ms (102 ± 25 versus 108 ± 38, *p* = 0.09), and the corrected QT interval (QTc) increased by 2 ms (454 ± 31 versus 456 ± 38, *p* = 0.65) among females but remained unchanged in males. In a subgroup analysis involving 16 patients with HFrEF, there was a significant increase in the QRS duration from 109 ± 22 ms to 120 ± 24 ms after the initiation of empagliflozin (*p* = 0.04). In a further subgroup analysis, the difference persisted only among the 10 patients on the 10 mg dose [75]. In conclusion, in patients with HF who receive empagliflozin 10 mg, the difference in QRS interval elongation is statistically significant. Therefore, empagliflozin showed no improvement in VD. However, the number of patients is anecdotal, and further studies are still awaited [76,77,78,79].

In addition, SGLT2i has demonstrated a significant role in reversing cardiac remodeling, which is a crucial process in improving cardiac function and prognosis in patients with HF, by reducing cardiac volumes, hypertrophy, and fibrosis, and improving overall cardiac energy efficiency and function [80].

Savage et al. [81] conducted a systematic review and meta-analysis including seven randomized, placebo-controlled trials in patients with HF comprising a total population of 657 patients. Outcome measures included left-ventricular end-diastolic volume and volume index (LVEDV/LVEDVi), left-ventricular end-systolic volume and volume index (LVSDV/LVSDVi), LVEF, left-ventricular mass index (LVMi), left-atrial volume index (LAVi), and left-ventricular global longitudinal strain (LV GLS). Pooled data demonstrated SGLT2 inhibition, compared with placebo control, resulted in significant improvements in the mean difference of LVEDV (*p* = 0.0004), LVEDVi (*p* = 0.002), LVESV (*p* = 0.0002), LVESVi (*p* = 0.006), LVM (*p* = 0.02), LVMi (*p* = 0.05), and LVEF (*p* = 0.0005). No significant difference in GLS (*p* = 0.18) or LAVi (*p* = 0.20) was noted.

It is plausible that the beneficial effects of dapagliflozin on VD may be mediated, at least in part, by its positive effects on reducing or preventing myocardial fibrosis. In preclinical studies, dapagliflozin has shown a potential effect on reducing cardiac fibrosis by inhibiting fibroblast activation [76,77,78,79]. Further clinical research is necessary to investigate this relationship and elucidate the underlying mechanisms in greater detail.

## 5. Vericiguat

Vericiguat is a soluble guanylate cyclase (sGC) stimulant that acts through a binding site independent of nitric oxide (NO), and it sensitizes sGC to endogenous nitric oxide by stabilizing nitric oxide binding to the binding site. HF is associated with an impairment in the synthesis of NO and decreased activity of sGC [82]. The “Vericiguat Global Study in Subjects With Heart Failure With Reduced Ejection Fraction” (VICTORIA) trial randomized 6857 patients with HFrEF (<45%) with a mean age of 67 years, 24% of whom were women, to receive either vericiguat or a placebo in addition to guideline-based medical therapy. The primary outcome was a composite of death from cardiovascular causes or first hospitalization for HF. Over a median of 10.8 months, a primary-outcome event occurred in 897 of 2526 patients (35.5%) in the vericiguat group and in 972 of 2524 patients (38.5%) in the placebo group (*p* = 0.02). A total of 691 patients (27.4%) in the vericiguat group and 747 patients (29.6%) in the placebo group were hospitalized for HF. Death from cardiovascular causes occurred in 414 patients (16.4%) in the vericiguat group and in 441 patients (17.5%) in the placebo group. The composite of death from any cause or hospitalization for HF occurred in 957 patients (37.9%) in the vericiguat group and in 1032 patients (40.9%) in the placebo group (*p* = 0.02). There are no subgroup analyses from this pivotal study or observational studies on the role of vericiguat in wide QRS.

Table 1 summarizes the trials about the efficacy of drugs for HF with wide QRS.

## 6. Conclusions

The presence of a wide QRS complex in HF patients remains a significant prognostic indicator, necessitating a comprehensive understanding of both its clinical implications and the evolving therapeutic landscape. While CRT remains a cornerstone in treating selected patients, the emergence of novel pharmacological agents, particularly sacubitril/valsartan and empagliflozin, has revolutionized HF management, offering additional avenues for improving outcomes in patients with both preserved and reduced ejection fraction, regardless of QRS duration. Future research should focus on further elucidating the complex interplay between electrical and mechanical dyssynchrony, optimizing patient selection for CRT in the era of new drug therapies, and exploring the potential synergistic effects of combining these innovative approaches to further mitigate the burden of heart failure in this challenging patient population.

## Figures and Tables

**Table 1:** Trials about the efficacy of drugs for heart failure with wide QRS.

Authors	Patients	Outcome	Results
Huang et al. [67] 2021	1168	Investigate the prescription patterns of sacubitril/valsartan among Taiwanese patients with heart failure with reduced ejection fraction	Multivariate analyses showed that wide QRS durations were negatively associated with the reverse cardiac remodeling
Allam et al. [66] 2024	100	Effects of sacubitril/valsartan on electrocardiogram indices and how those parameters relate to echocardiographic parameters	QRS width was reduced from 123.7 ± 20.3 to 117.1 ± 18.8 ms (*p <* 0.001)
Abudan et al. [65] 2022	368	The clinical impacts of sacubitril/valsartan on reverse cardiac remodeling in patients di HFrEF with different QRS durations	During a median follow-up of 9.1 months, five resolved LBBB to normal conduction (*p* = 0.11), and two improved to non-specific intraventricular conduction delay (*p* = 0.08)
Abdin et al. [70] 2024	9824	The effect of dapagliflozin according to QRS duration across the spectrum of left-ventricular ejection fraction	The risk of the primary outcome consistently across the QRS duration subgroups (HR [95% CI] 0.75 [0.67–0.85], 0.79 [0.65–0.96], and 0.89 [0.70–1.13] in the <120, 120–149, and ≥150 ms groups, respectively; *p* for interaction = 0.28)

CI: confident interval; HFrEF: heart failure with reduced ejection fraction; HR: hazard ratio.
